# SERS Platform Based on Hollow-Core Microstructured Optical Fiber: Technology of UV-Mediated Gold Nanoparticle Growth

**DOI:** 10.3390/bios12010019

**Published:** 2021-12-31

**Authors:** Anastasiia A. Merdalimova, Polina G. Rudakovskaya, Timur I. Ermatov, Alexander S. Smirnov, Sergey S. Kosolobov, Julia S. Skibina, Polina A. Demina, Boris N. Khlebtsov, Alexey M. Yashchenok, Dmitry A. Gorin

**Affiliations:** 1 Center for Photonic Science and Engineering, Skolkovo Institute of Science and Technology, 3 Nobel str, 121205 Moscow, Russia; p.rudakovskaya@skoltech.ru (P.G.R.); timur.ermatov@skolkovotech.ru (T.I.E.); A.Yashchenok@skoltech.ru (A.M.Y.); 2 Center for Design, Manufacturing and Materials, Skolkovo Institute of Science and Technology, 1 Nobel str, 121205 Moscow, Russia; Al.Smirnov@skoltech.ru (A.S.S.); s.kosolobov@skoltech.ru (S.S.K.); 3 SPE LLC Nanostructured Glass Technology, 101 50 Let Oktjabrja str, 410033 Saratov, Russia; skibinajs@yandex.ru; 4 FSRC “Crystallography and Photonics” RAS, 59 Leninsky pr., 119333 Moscow, Russia; polidemina1207@yandex.ru; 5 Shemyakin-Ovchinnikov Institute of Bioorganic Chemistry RAS, Miklukho-Maklaya str. 16/10, 117997 Moscow, Russia; 6 Saratov Scientific Centre of the Russian Academy of Sciences (IBPPM RAS), Institute of Biochemistry and Physiology of Plants and Microorganisms, 410049 Saratov, Russia; khlebtsov_b@ibppm.ru

**Keywords:** hollow-core microstructured optical fiber, SERS, low-volume sensing, biosensing, gold nanoparticles, gold deposition, glass surface modification, ultraviolet reduction

## Abstract

Surface-enhanced Raman spectroscopy (SERS) is a powerful technique for biosensing. However, SERS analysis has several concerns: the signal is limited by a number of molecules and the area of the plasmonic substrate in the laser hotspot, and quantitative analysis in a low-volume droplet is confusing due to the change of concentration during quick drying. The usage of hollow-core microstructured optical fibers (HC-MOFs) is thought to be an effective way to improve SERS sensitivity and limit of detection through the effective irradiation of a small sample volume filling the fiber capillaries. In this paper, we used layer-by-layer assembly as a simple method for the functionalization of fiber capillaries by gold nanoparticles (seeds) with a mean diameter of 8 nm followed by UV-induced chloroauric acid reduction. We also demonstrated a simple and quick technique used for the analysis of the SERS platform formation at every stage through the detection of spectral shifts in the optical transmission of HC-MOFs. The enhancement of the Raman signal of a model analyte Rhodamine 6G was obtained using such type of SERS platform. Thus, a combination of nanostructured gold coating as a SERS-active surface and a hollow-core fiber as a microfluidic channel and a waveguide is perspective for point-of-care medical diagnosis based on liquid biopsy and exhaled air analysis.

## 1. Introduction

Surface-enhanced Raman spectroscopy (SERS) is a powerful tool for biological analysis, as it is based on Raman spectroscopy, which overcomes such issues of predominantly used fluorescent probes as limited multiplexing capability, poor photostability, and autofluorescence background [1]. In important polymeric biomolecules, such as proteins and nucleic acids, some specific subgroups are repeated many times, and therefore their vibrational modes may provide prominent Raman signals, such as amide modes from peptide groups [2]. However, in many cases, the Raman signal itself is too weak, as just few incident photons contribute to this inelastic scattering. Therefore, the SERS technique is introduced. Surface enhancement is based on a local surface plasmon resonance on metallic nanostructures, that provides amplification of any radiation, including Raman scattering, in the vicinity of the structure [3].

Fiber-optic SERS sensors utilize fiber to guide incident and scattered radiation from the sensing tip [4,5,6] or to be an integrating sensor itself with fiber walls modification [4,7]. Raman and SERS sensing have the following features: Raman signal intensity for a given liquid sample and measurement conditions depends on the irradiated number of molecules; the surface enhancement factor provided by a given SERS substrate depends on the area of the plasmonic surface irradiated. These challenges can be improved by using optical fibers as Raman/SERS platforms [8,9,10]. The fiber used may represent a capillary [9] or a microstructured optical fiber (MOF) with a hollow [10,11,12] or a solid core [13,14]. For MOFs, hollow-core microstructured optical fibers (HC-MOFs) have the advantage of reduced background Raman signal from the fiber material (glass), as the light is confined in the hollow core. The structure of HC-MOFs [10,15,16,17] is defined by a hollow core surrounded by a microstructured cladding array running along the entire fiber length [7], which enables transmission phenomena that will be discussed and exploited further. Moreover, the utilization of fibers as Raman sensors can solve the problem of unreliable quantitative measurements in drying drops, where sample concentration and focusing may change rapidly during measurement due to drying [16]. Meanwhile, HC-MOFs allow the work in a low sample volume [12], for example, a fiber with a hollow core diameter of 240 µm and length of a few centimeters is filled with just a few microliters for analyte probing.

An appropriate material for SERS enhancement, according to the Drude model, should fit excitation wavelength: firstly, a real part of dielectric function −20 ≤ Re(ε) ≤ −1, implying refractive index (RI) < 1 and high reflection; secondly, small imaginary part Im(ε), that means low absorption. According to these requirements, silver is suitable for SERS in the entire visible range. However, considering technological aspects, such as durability and toxicity, gold is more preferable while using wavelength >600 nm, where its optical properties also meet the requirements [18].

There are different approaches to providing SERS inside HC-MOFs. Plasmonic nanostructures with the analyte may be in a colloidal solution [11,14] or localized on fiber walls [12,19,20,21,22]. Several material combinations have been used for building SERS substrate on fiber walls, among others, such as graphene oxide with poly(diallyl dimethylammonium chloride) (PDDA) and silver nanoparticles (AgNPs) [20], (3-mercaptopropyl)trimethoxysilane(MPTMS) and gold nanoparticles (AuNPs) [21], polycationic poly(allylamine hydrochloride) (PAH), and polyanionic poly(styrene sulfonate) (PSS) multiple layers with AuNPs [16]. Meanwhile, the amount of plasmonic (gold or silver) nanoparticles loaded in such systems is undetermined. For planar and colloidal nanostructures, it is known that chloroauric acid reduction on seeds may be used with a reducing agent [23,24] or a reducing agent and UV irradiation [25,26,27,28], which is beneficial in our case to catalyze the reaction just when the solution is inside the HC-MOF.

In this paper, we developed SERS substrate using UV-mediated chloroauric acid reduction on gold seeds, firstly on planar glass slides and then inside HC-MOFs. We also introduced a method for control of SERS substrate deposition inside HC-MOF, according to changes in transmission spectra of HC-MOF.

## 2. Materials and Methods

### 2.1. Chemical Reagents and Equipment for Fabrication

Polyethyleneimine (PEi, MW 750,000), chloroauric acid (HAuCl4, 1% aqueous solution), and trisodium citrate (Na3C6H5O7, 1% aqueous solution) were purchased from Sigma-Aldrich. For PEi dilution and sample wash, deionized water (18 MΩ × m, Millipore) was used. For HC-MOF pump coating, a peristaltic pump (Shenchen, China) and a flexible silicone tube with an inner diameter of 1 mm were used.

**Figure 1:**
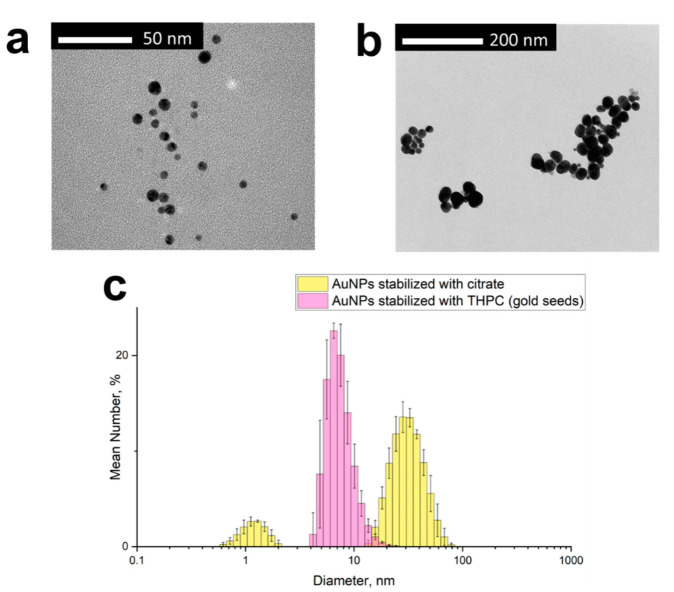
Characterization of AuNPs: (**a**) transmission electron microscopy (TEM) image of THPC gold seeds; (**b**) TEM image of AuNPs stabilized by citrate (yellow color); (**c**) size distribution histogram of gold seeds stabilized by THPC and AuNPs stabilized with citrate (pink color), revealed by dynamic light scattering (DLS).

AuNP seeds, which are an aqueous solution of AuNPs with tetrakis-(hydroxymethyl)-phosphonium chloride (THPC) as simultaneously a reducing agent and stabilizing ligand, were synthesized as described previously [29], with gold concentration 1 mg/mL. Three months passed between the seed preparation and their utilization in the current experiment, and therefore their size increased due to Ostwald ripening. Larger AuNPs stabilized by citrate in an aqueous solution were synthesized by a modified Turkevich method [30], with gold concentration 0.2 mg/mL. Both types of AuNPs were imaged by transmission electron microscopy (TEM), and their size distribution was estimated using dynamic light scattering (DLS), as demonstrated in Figure 1. TEM images were taken on a Zeiss M912 Omega transmission electron microscope (Carl Zeiss Microscopy GmbH, Oberkochen, Germany) at an operating voltage of 300 kV. DLS analysis was provided on Zetasizer Nano ZS (Malvern Panalytical), with 3 measurements for each sample.

As a UV light source, Camelion LH26-FS 26 W light bulb was used, with an emission spectrum centered at 365 nm. Samples were placed approximately 3 cm under the vertically mounted bulb, so that power density on the sample measured at 365 nm was 4.3 mW/cm^2^.

### 2.2. SERS Substrate Fabrication on Planar Slides

Firstly, UV-mediated chloroauric acid reduction on gold seeds was developed on planar glass slides. For that, objective glass slides underwent 2 min plasma treatment. Then, slides were incubated for 7 min with PEi at concentration 2 mg/mL in deionized water under continuous stirring. Afterward, the slides were rinsed for 2 min with deionized water. Further, the slides were incubated overnight: the first group of slides with citrate AuNPs, and the second control group with THPC AuNP seeds. The concentration of gold in AuNPs stabilized with citrate used was 0.2 mg/mL, which provided a volume fraction 1.04%; the gold seeds stabilized with THPC had a concentration of 0.25 mg/mL (4× diluted from the initial value), providing a volume fraction of 1.23%. Therefore, the volume concentrations of both types of AuNPs were quite close to each other. Both types of AuNPs were deposited overnight (14 h) under continuous stirring. Furthermore, slides that were incubated with THPC gold seeds were covered with an approximately 2 mm layer of a mixture of 0.01% chloroauric acid and 0.01% trisodium citrate in 2:1 *v*/*v* and irradiated by a UV lamp for 15 min, and then finally gently washed with water.

### 2.3. SERS Substrate Fabrication in HC-MOF

The hollow-core microstructured optical fibers that were used have a hollow core diameter at around 240 um and three surrounding capillary layers. Spectral properties of such HC-MOF have been described previously [31,32,33].

The protocol for deposition and its control is described in a step-by-step manner in the Supplementary Materials. Briefly, the deposition process was performed as follows: firstly, every fiber underwent 2 min plasma treatment, and its transmission spectra were measured. Then, PEi at concentration 2 mg/mL was delivered for 7 min at a speed of 150 mL/min using a peristaltic pump, as has been described previously [31]. Afterward, HC-MOF was washed for 2 min by deionized water and dried with air flow using a syringe; then, an hour later, the transmission spectrum was measured. Following this, THPC AuNP seeds at concentration 0.1 mg/mL (10× diluted from the initial value), providing volume fraction 0.5%, were deposited overnight (14 h) at a speed of 150 µL/min using the same pumping system. Afterward, HC-MOF was washed for 2 min with deionized water and dried with air flow using a syringe; then, an hour later, the transmission spectrum was measured. Furthermore, HC-MOF was filled with a mixture of 1% chloroauric acid and 1% trisodium citrate in 2:1 *v*/*v* and irradiated by a UV lamp for 2 h, being left in a vial with the solution to avoid drying. Finally, HC-MOF was dried at 50 °C overnight and washed with water, then dried with air flow using a syringe, and an hour later, the transmission spectrum was measured.

### 2.4. Transmission Spectra of HC-MOFs

To verify the adsorption of functional materials onto fiber capillaries, we measured the optical transmission of functionalized fibers since the formation of nanocoating leads to the growth in the effective thickness of capillary walls and shifts in the fiber transmission windows towards longer wavelengths [31].

The optical setup used in our experiments was described by Ermatov et al. [31]. The output light of a broadband halogen lamp Thorlabs SLS201L (Thorlabs, Newton, NJ, USA, 360–2600 nm) was initially collimated Thorlabs F220SMA-532 (Thorlabs, Newton, NJ, USA) and then focused by a 10× objective (Olympus Tokyo, Japan) to the HC-MOF input. The other 10× objective (Olympus, Tokyo, Japan) was used to collect the transmitted light, which was further guided to a compact CCD spectrometer Thorlabs CCS100 (Thorlabs, Newton, NJ, USA) operating in the extended wavelength region (200–1000 nm). All of the transmission spectra were initially normalized to the spectrum of the lamp and then to its maximum value [31]. Positions of minima were calculated using a centroid function.

### 2.5. Raman and SERS Measurements

As a model analyte, Rhodamine 6G (R6G) aqueous solution was used at a concentration of 0.2 mM for SERS measurements and additionally at a concentration of 20 mM for Raman measurements at planar slides. Raman and SERS measurements were provided on Raman spectrometer LabRAM HR Evolution (HORIBA France SAS, Longjumeau, France), equipped with a diffraction grating 600 lines/mm, objective Olympus MPlan 10×, 633 nm laser at 12 mW power, and 2 s exposure time; 5 accumulations were used. All spectra were corrected for baseline using built-in Labspec 6 software. For mean +/− standard deviation representation, 5 measurements of the same sample were averaged.

For Raman and SERS measurements in HC-MOF, a backscattering signal was used while focusing on the center of the fiber end face, with an optical axis parallel to fiber. Two types of HC-MOF were used for measurements: bare and coated with a two-step gold deposition.

### 2.6. SEM Analysis

SEM observations were carried out without a metal coating using JEOL JSM-7200F FE-SEM. The hollow-core microstructured fibers (HC-MOFs) were analyzed at 30 keV accelerating voltage using a SE-detector. For the observation of the SERS-active substrates on glass slides, a beam deceleration function and charge-free scanning mode was used. To reduce the charging phenomenon, we decelerated the beam in the vicinity of the specimen to 1.5 keV (gentle-beam mode). To use the low acceleration voltage, the sample was biased to 2.0 kV. SEM images were taken by using an upper detector (UED) with the backscattered electron mode.

## 3. Results and Discussion

This study aimed at developing a novel approach for the easy preparation of SERS substrate inside HC-MOF. For that, firstly we proved a concept on glass slides. We prepared planar SERS substrates using a two-step UV-mediated chloroauric acid reduction on gold seeds and compared their SERS enhancement with the substrates made by one-step absorption of larger AuNPs. This comparison demonstrated the efficiency of the developed two-step method. Then, we transferred the technology to HC-MOFs. We also introduced a method for SERS substrate deposition process control in HC-MOF, based on the change in its transmission spectra.

### 3.1. Fabrication of SERS Substrates on Planar Slides

Firstly, we fabricated SERS active substrates on planar objective slides. For that, two types of AuNPs were compared: small AuNPs (gold seeds) stabilized with THPC [29] and larger AuNPs stabilized with citrate, synthesized by a modified Turkevich method [30]. TEM and DLS characterization of these two types of AuNPs are provided in Figure 1. According to DLS (Figure 1c), gold seeds have a mean diameter and standard deviation of 8.0 nm and 1.4 nm, respectively; AuNPs stabilized with citrate have a mean diameter and standard deviation of 33 nm and 11 nm, respectively. However, according to DLS, AuNPs stabilized with citrate have a fraction with a size of around 1 nm. TEM image (Figure 1b) also contains a few objects much smaller in size than the main fraction. We assume they are citrate gold seeds.

**Figure 2:**
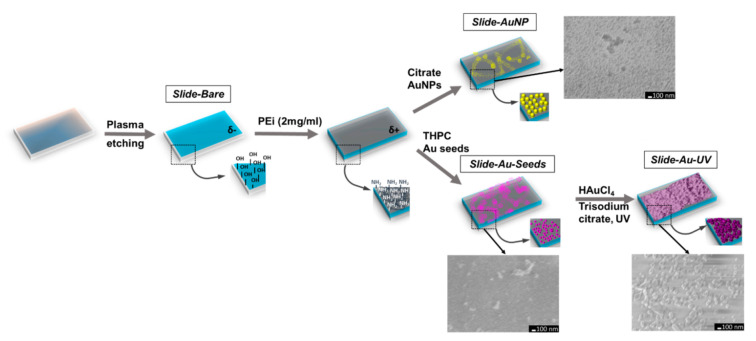
The fabrication process of SERS-active substrates on glass slides with scanning electron microscopy (SEM) images incorporated. The scale bar is 100 nm.

To compare Raman and SERS signal, we fabricated three types of samples, as illustrated in Figure 2: bare glass slide (sample Slide-Bare); glass slide functionalized with PEi and citrate-stabilized AuNPs (sample Slide-AuNP); glass slide functionalized with PEi and UV-mediated chloroauric acid reduction on gold seeds (sample Slide-Au-UV). Initially, objective glass slides were taken and etched with plasma to clean the surface and to obtain hydroxyl groups on the glass surface. Then, slides were covered with PEi to obtain a positive surface charge, which is necessary for further efficient electrostatic absorption. Then two types of negatively charged AuNPs (stabilized with citrate and with THPC) were adsorbed on slides by incubation and thus samples Slide-AuNP and Slide-Au-Seeds were obtained, respectively. The last step was Slide-Au-Seeds improvement to Slide-Au-UV by UV-mediated reduction of HAuCl4 in citrate presence.

As it can be noticed from SEM images incorporated into Figure 2, the sample Slide-Au-Seeds was covered with small AuNPs and in some places with their aggregates. The sample Slide-AuNP was covered with larger AuNPs more intensively, although gold mass concentration here was of the same order as in Slide-Au-Seeds, and taking into account particle sizes, we found that concentration by particle number was even less than for the sample Slide-Au-Seeds. After UV-mediated gold reduction, the obtained sample Slide-Au-UV had a significant increase in the size of gold nanostructures on its surface compared to the previous state on the sample Slide-Au-Seeds.

### 3.2. SERS Substrates on Planar Slides: SERS Measurements

To prove Raman signal enhancement, we used R6G as a model Raman reporter [34,35,36]: 2 µL of 0.2 mM R6G aqueous solution was placed dropwise on the prepared substrates. For a bare slide, a 100x greater concentration of R6G was also probed.

**Figure 3:**
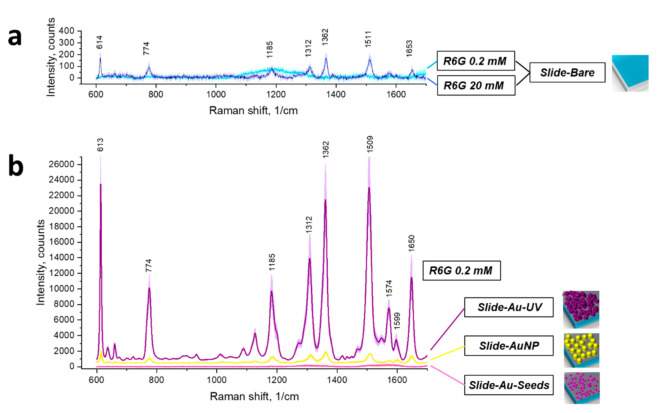
Raman and SERS of Rhodamine 6G (R6G) on planar glass substrates: (**a**) R6G at concentrations 0.2 and 20 mM on a bare glass slide; (**b**) R6G at concentration 0.2 mM on plasmonic substrates.

According to the acquired spectra presented in Figure 3, on a bare glass slide, the Raman signal of 0.2 mM R6G was not detected; meanwhile, 20 mM R6G provided a distinguishable specific Raman fingerprint. Raman and SERS intensities of the most prominent R6G peaks are described in Table S1. For a simple substrate comparison, an analytical enhancement factor (EF) was calculated as follows [37]:

(1) EF=ISERScSERSIRcR,

where $c_{R},{\;c}_{\mathrm{SERS}}$ are R6G concentrations during Raman (at a bare substrate) and SERS measurements, and $I_{R},{\;I}_{\mathrm{SERS}}$ are their intensities, respectively, pairwise for each Raman peak considered. In Table S1, EF is calculated for each pair of prominent R6G Raman peaks detected. For a general EF estimation for the substrates, Raman modes at 1362 and 1509 cm^−1^ were used, as they correspond to benzene ring stretching and are the most prominent ones. The sample Slide-Au-Seeds did not demonstrate any signal from 0.2 mM R6G. The sample Slide-AuNP formed by 33 nm citrate-stabilized AuNPs demonstrated EF around 0.8 × 10^3^; meanwhile, the sample Slide-Au-UV, using UV-mediated chloroauric acid reduction, demonstrated EF 1.3 × 10^4^.

Therefore, UV-mediated chloroauric acid reduction on gold seeds demonstrated its potential in improving SERS signal, and this approach was adopted to HC-MOF, due to the ability of HC-MOF to integrate signal across the entire length with low-volume sensing and to mitigate a problem of sample volume change during measurements [12,16,38].

### 3.3. SERS Substrates in HC-MOF: Fabrication and Layer Deposition Control

**Figure 4:**
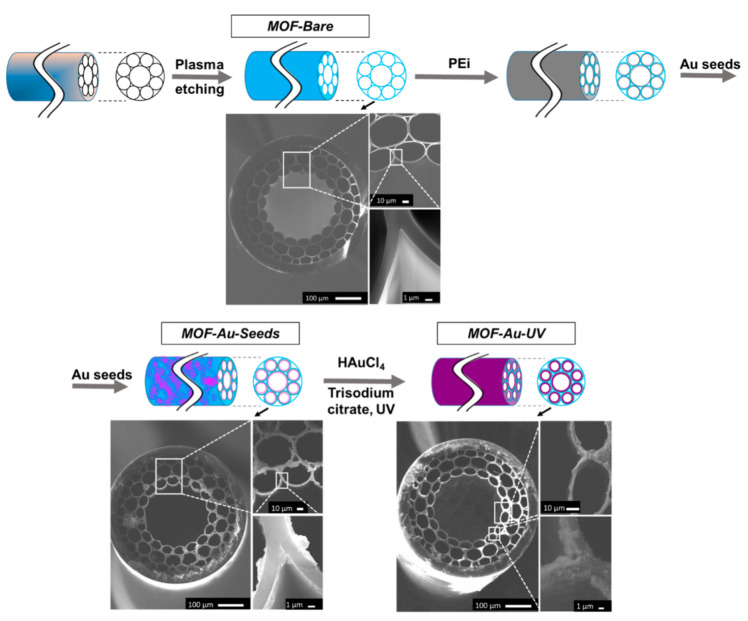
The fabrication process of SERS-active HC-MOFs with SEM images incorporated.

Sample fabrication is illustrated in Figure 4. Similar to the technology used for slides, firstly, HC-MOFs were treated with plasma to clean and to obtain hydroxyl groups on fiber walls. Then, HC-MOFs were functionalized with PEi using a peristaltic pump to obtain a positive surface charge, which is necessary for further efficient electrostatic absorption. Following this, using the same peristaltic pump, we electrostatically adsorbed AuNP seeds stabilized with THPC, and thus the sample MOF-Au-Seeds was obtained. The last step was MOF-Au-Seeds improvement to MOF-Au-UV by UV-mediated reduction of HAuCl_4_ in the presence of citrate.

SEM images of the fiber tip on different steps of the functionalization are incorporated into Figure 4. Additionally, the SEM images of the tip and middle sections of the fabricated HC-MOF sensors are provided in the Supplementary Materials in Figure S1, and photographic images for visual inspection are demonstrated in Figure S2. Both in Figures S1 and S2, a difference between results of surface modification on the tip and in the middle may be noticed: in the middle, deposition is present, but is less intense.

While developing a novel technology for HC-MOF-coating, one needs to evaluate performance. Layer deposition control using SEM images has several disadvantages. One image provides information about only one cross-section at the moment, on the examined fiber tip. As it was demonstrated, the SEM imaging of the tip is not representative, and fibers need to be broken up for coating control. Besides this, SEM equipment is expensive, its usage is laborious and requires special skills, and it is usually not routinely available. Furthermore, since most of the commercial fibers are made of silica and its counterparts, an extra deposition of conductive metals required for the proper imaging decreases the contrast between fiber walls and functional coating and does not allow for resolving nanometer-thin films [39]. An optical setup for transmission spectra measurement of HC-MOFs [31] solves this problem: spectral data provide integral information about HC-MOF geometry. Moreover, this optical setup is quite compact, is quickly mounted from common optical components, and is simple in use. The mechanism of light guidance in the employed HC-MOFs can be described by the model of Fabry–Perot resonances and antiresonances in the wall of the central capillary [32,33].

**Figure 5:**
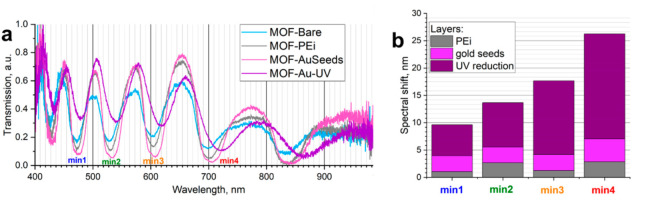
Shifts in transmission spectra induced by coatings: (**a**) transmission spectra of a single HC-MOF at all stages of layer deposition, with positions of minima selected for analysis labeled as min1 … min4; (**b**) shifts in transmission spectra induced to 1 fiber cumulatively by PEi, gold seeds, and UV-mediated chloroauric acid reduction.

Figure 5a demonstrates transmission spectra for a single HC-MOF at all stages of the surface functionalization. For quantitative analysis, positions of minima min1 … min4 were chosen, as earlier and later spectral components have lower signal-to-noise ratios, which may affect the accuracy of minima position determination. In Figure 5b, calculations of shifts in positions of transmission minima are presented for all stages of layer deposition cumulatively. It is worth noting that these shifts depend on additional optical path lengths experienced by a divergent beam inside the HC-MOF, and the optical path length depends both on refractive index and layer thickness. However, with wavelength increasing, the distance between minima becomes greater in a single spectrum, and the same optical path lengths lead to greater shifts in transmission spectra. This phenomenon compensates (and even overcompensates) the fact that the refractive index of gold in the visible range is mainly decreasing with increasing wavelength [40].

Furthermore, PEi coating was briefly studied. Transmission spectra of four samples before and after PEi coating were taken, and PEi-induced spectral shifts were extracted, as illustrated in Figure S3. Here, sample 1 was measured 19 days after PEi deposition; sample 2 was measured 9 days after PEi deposition, and measurement 2* is the same sample measured 1 h after deposition; samples 3 and 4 were also measured 1 h after deposition. The sample from Figure S3a may be found on Figure S3b under label 4. Error bars correspond to the standard deviation of the minima position, calculated once by placing it to the optical system and re-adjusting one bare HC-MOF 30 times. It can be noticed that “old” samples, measured quite a long time ago after PEi deposition, demonstrated lower shifts in transmission spectra, which implies lower additional optical path induced. Optical path length depends on thickness and refractive index. The observed effect of spectral shift decrease with time may be explained by the presence of embedded water and PEi dehydration with time [41]. During this process, thickness decreases, but refractive index increases, as PEi refractive index in the visible range is around 1.6 [42], while the refractive index of water is around 1.33. Therefore, it seems we watch that layer thickness decreases with time stronger than refractive index increases.

### 3.4. SERS Substrates in HC-MOF: SERS Measurements

**Figure 6:**
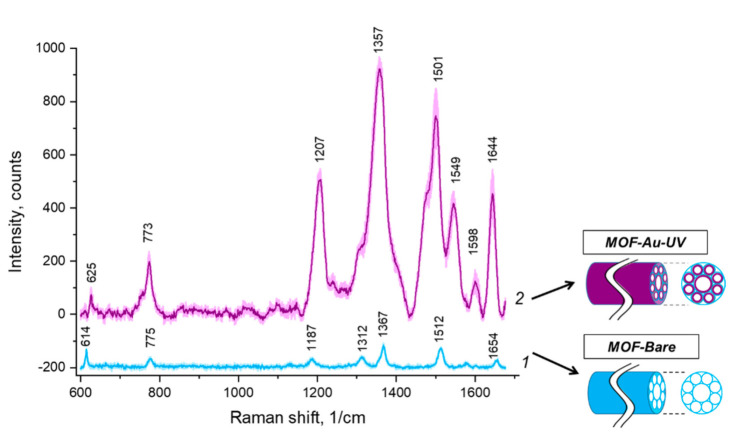
Raman and SERS of R6G in HC-MOF: 1—bare HC-MOF; 2—HC-MOF with UV-mediated chloroauric acid reduction on gold seeds.

Raman and SERS spectroscopy of R6G in HC-MOF was studied (Figure 6, Table S2), comparing a bare HC-MOF and HC-MOF coated with the proposed UV-mediated chloroauric acid reduction on gold seeds, respectively. Functionalized HC-MOF demonstrates SERS enhancement with an analytical EF around 10x.

Firstly, comparing Figure 3 and Figure 6, one may conclude that using HC-MOF is beneficial for Raman intensity: R6G with concentration 0.2 mM is distinguishable in HC-MOF; meanwhile, it was not found on planar slides. If we were to calculate “fiber analytical Raman enhancement factor” analogously to analytical SERS enhancement factor introduced in Equation (1), we would obtain 50× enhancement. This may be explained by a larger effective volume of analyte undergoing Raman scattering due to waveguiding properties of HC-MOF. Meanwhile, the large height of liquid analyte in HC-MOF is provided by a sample volume of around 1.1 µL in the hollow core with 2.5 cm length.

Secondly, the analytical EF for the present SERS substrate in HC-MOF is around 10×, which is lower than for the SERS substrate with the same fabrication technology but on planar slides. We propose that although the SERS substrate area reached by laser irradiation in HC-MOF is greater than on planar substrate, the efficiency of scattering signal collection by an objective is lower in this case. Nanostructured gold coating on inner fiber walls does not only enhance Raman scattering signal but scatters incident radiation in multiple directions at the same moment. Therefore, compared to the planar substrate irradiation, a smaller part of the generated signal (only backscattered to objective aperture) is collected by the Raman spectrometer. This problem may be solved by installing a mirror on the fiber tip opposite to the illumination and signal collection side. Furthermore, as could be noticed in Figure 5, initially HC-MOFs were chosen so that laser wavelength of 633 nm is around the maximum of the fiber transmission. However, after UV-mediated gold reduction, the HC-MOF transmission spectrum shifted in such a way that 633 nm is around the minimum of the fiber transmission, which is not an optimal choice and further studies are needed on HC-MOFs that possess maximum transmission on 633 nm after SERS substrate formation. Moreover, further improvement of the developed technology of gold deposition to HC-MOF may be provided. In particular, the developed UV-mediated gold reduction in HC-MOF could be combined with further annealing [8].

## 4. Conclusions

A SERS platform based on HC-MOF was fabricated using UV-mediated chloroauric acid reduction on gold seeds deposited to the inner hollow core surface preliminarily modified by PEi. Firstly, the superiority of such technology was proved on planar substrates: compared to a SERS substrate based on the one-step deposition of AuNPs with greater size, the developed SERS substrate based on two-step gold deposition (UV-mediated chloroauric acid reduction on gold seeds) provided 20× greater SERS enhancement. Therefore, for HC-MOFs, modification by gold seeds was chosen followed by chloroauric gold reduction.

In the HC-MOF-based sensor, SERS enhancement was also observed, but at a lower rate; therefore, the deposition technology may be further improved to reach enhancement factor obtained on the plain SERS substrate fabricated using a similar approach.

Furthermore, HC-MOF has a quasi-sinusoidal transmission spectrum in the visible range. On the basis of this, we introduced a simple and quick spectroscopic control of SERS-active substrate fabrication inside HC-MOF. Coatings induce shifts due to extra optical pathlength experienced by a divergent beam. In the present article, tracing shifts of minima in transmission spectra was demonstrated as a feasible way to control subsequent layer deposition. Additionally, a study of PEi coating with the presented method revealed its thinning with time, which correlates to the fact of embedded water presence in PEi and further dehydration.

Thus, SERS platforms based on HC-MOF modification using UV-mediated chloroauric acid reduction on gold seeds were fabricated. Analysis of shifts in HC-MOF transmission spectra was used for layer deposition control. This feature can be used for in situ quality control during the fabrication of such type of SERS platform. The same approach could be used for analyte amount evaluation in the case of an analyte capable to form a monolayer on the prepared gold nanostructured SERS coating. Such platforms are perspective as biosensors for liquid biopsy and exhaled gas analysis.

## Supplementary Materials

The following supporting information can be downloaded at: https://www.mdpi.com/article/10.3390/bios12010019/s1, Table S1. R6G Raman and SERS peaks assignment and enhancement factor (EF) for SERS substrates on planar slides. Table S2. R6G Raman and SERS peaks assignment and enhancement factor (EF) for SERS substrates on Hollow-Core Microstructured Optical Fiber (HC-MOF). A protocol for Step-by-step process of HC-MOF SERS substrate fabrication. Figure S1. SEM inages of the tip, end and middle sections of the fabricated HC-MOF sensors. Figure S2. Macro photos of HC-MOFs on different stages of SERS substrate fabrication. Figure S3. Shifts in transmission spectra induced by PEi: (a) transmission spectra of the same HC-MOF before and after PEi deposition, with positions of minima selected for analysis labeled as min1 … min4; (b) shifts in transmission spectra induced by PEi to a set of HC-MOFs.
