# Supplementary material for: SERS Platform Based on Hollow-Core Microstructured Optical Fiber: Technology of UV-Mediated Gold Nanoparticle Growth
Source: Biosensors (Basel). 2021 Dec 31;12(1):19. doi: 10.3390/bios12010019 (PMC8774134; doi:10.3390/bios12010019)
Supplement: Supplementary file 1 [file biosensors-12-00019-s001.zip › biosensors-1498547-supplementary.pdf]

biosensors

MDPI

Communication

SERS Platform Based on Hollow-Core Microstructured Optical Fiber: Technology of UV-Mediated Gold Nanoparticle Growth

Anastasiia A. Merdalimova [1,*], Polina G. Rudakovskaya [1], Timur I. Ermatov [1], Alexander S. Smirnov [2], Sergey S. Kosolobov [2], Julia S. Skibina [3], Polina A. Demina [4,5], Boris N. Khlebtsov [6], Alexey M. Yashchenok [1] and Dmitry A. Gorin [1,*]

Table S1. R6G Raman and SERS peaks assignment and enhancement factor (EF) for SERS substrates on planar slides.

Raman shift, cm^{-1}	Peak assignment [1]	Intensity, counts			EF	
		R6G, 20 mM	Slide-AuNP + R6G 0.2 mM	Slide-Au-UV + R6G 0.2 mM	Slide-AuNP	Slide-Au-UV
613	C–C–C ring in-plane bending	169	1381	22492	817	13309
775	C–H out-of-plane bending	99	569	9124	575	9216
1185	C–H in-plane bending	80	762	8726	953	10908
1312	aromatic C–C stretching	97	972	12814	1002	13210
1362	aromatic C–C stretching	163	1392	20497	854	12575
1509	aromatic C–C stretching	160	1180	22051	738	13782
1574	aromatic C–C stretching	48	289	6405	602	13344
1650	aromatic C–C stretching	79	823	10502	1042	13294

Table S2. R6G Raman and SERS peaks assignment and enhancement factor (EF) for SERS substrates on Hollow-Core Microstructured Optical Fiber (HC-MOF). A protocol for Step-by-step process of HC-MOF SERS substrate fabrication.

MOF-Bare + R6G		MOF-Au-UV + R6G		EF
Raman shift, cm^{-1}	Intensity, counts	Raman shift, cm^{-1}	Intensity, counts	
613	67	625	950	14,2
775	34	773	197	5,8
1183	33	1207	506	15,3
1312	41	-	-	-
1364	85	1357	923	10,9
1512	71	1501	747	10,5
1654	26	1644	453	17,4

Step-by-step process of HC-MOF SERS substrate fabrication

1. Transmission spectrum measurement of initial 6 cm HC-MOFs;
2. Plasma treatment for 2 min to clean and activate surfaces;
3. PEi delivery at concentration 2 mg/mL for 7 min at speed 150 mL/min using a peristaltic pump;
4. 2 min wash by deionized water;
5. Air blowing with a syringe and air dry at room temperature;
6. Transmission spectrum measurement;
7. THPC AuNP seeds deposition overnight (14h) at speed 150 mL/min using a peristaltic pump;
8. 2 min wash by deionized water;

9. Air blowing with a syringe and air dry at room temperature;
10. Transmission spectrum measurement;
11. Cutting HC-MOFs to 2 pieces with length 2.5-2.8 cm;
12. Transmission spectrum measurement, as it may differ in cut pieces compared to initial fibers due to nonuniformity in layer formation;
13. Capillary filling with a mixture of 1% chloroauric acid and 1% trisodium citrate in 2:1 *v/v* and irradiation by a UV lamp for 2 h, being left in a vial with the solution for continuous solution supply during evaporation;
14. Drying at 50 C overnight;
15. Water washing and air blowing with a syringe;
16. Transmission spectrum measurement.

Here, steps of drying and transmission measurement (1, 5-6, 9-10, 12, 16) are additional steps for layer deposition control. Steps 11-12 related to fiber cutting are optional, as allow to get more samples for independent UV irradiation where parameters may be varied for technology optimization. However, the samples should be cut somewhen before Raman/SERS measurement, as 2.8 cm is a maximum size that is technologically suitable to mount to the available backscattering Raman spectrometer setup.

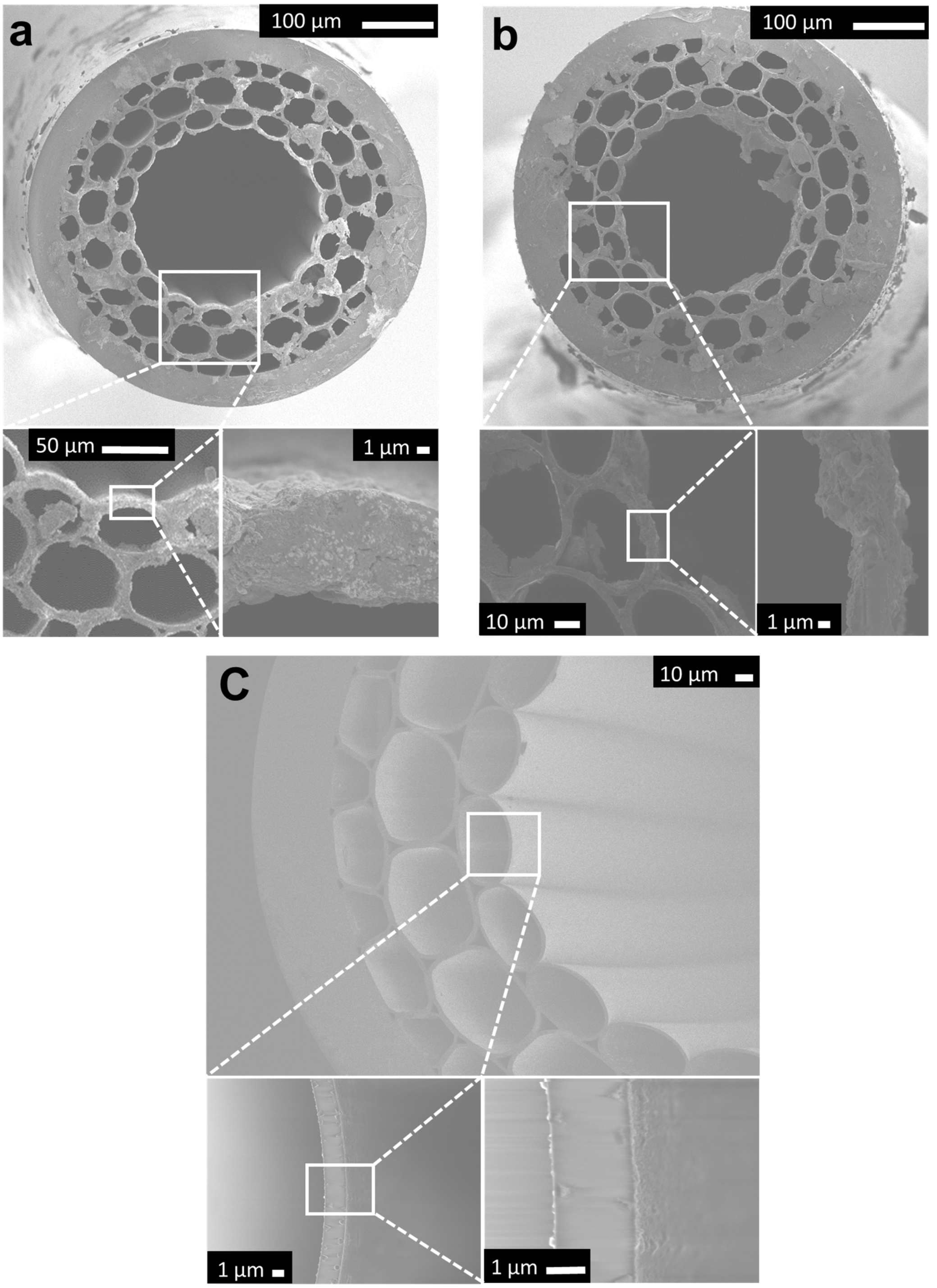

Figure S1. SEM images of the tip, end and middle sections of the fabricated HC-MOF sensors.

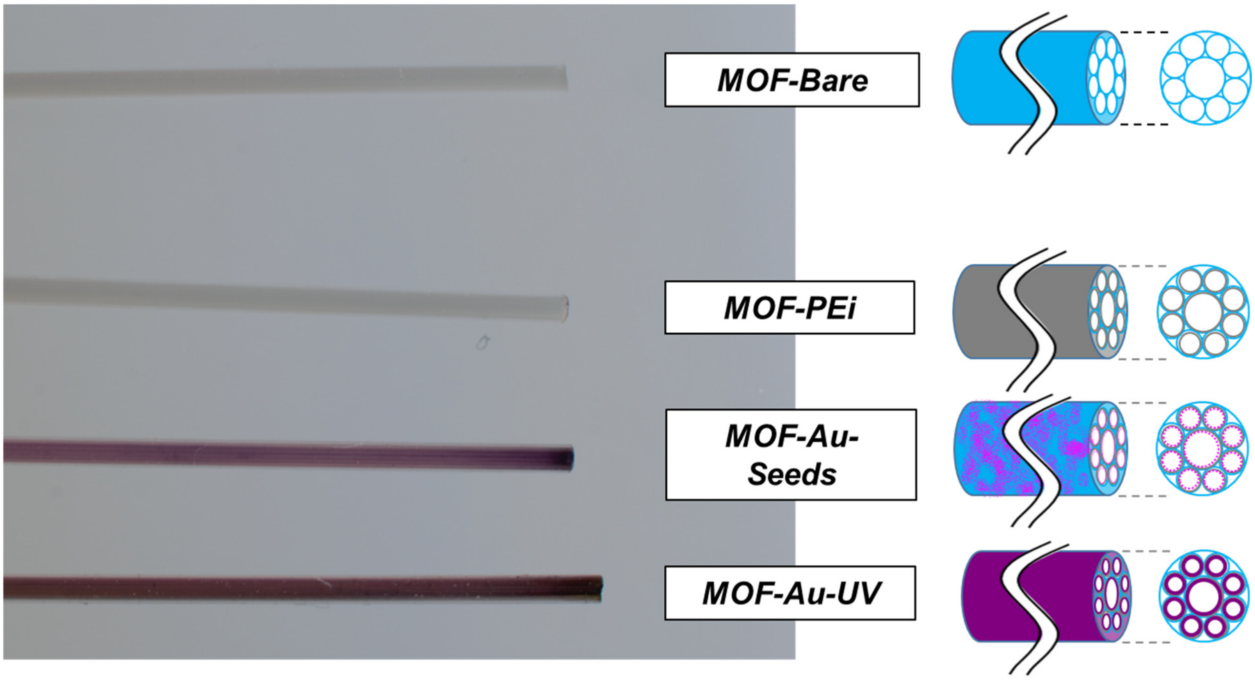

Figure S2. Macro photos of HC-MOFs on different stages of SERS substrate fabrication.

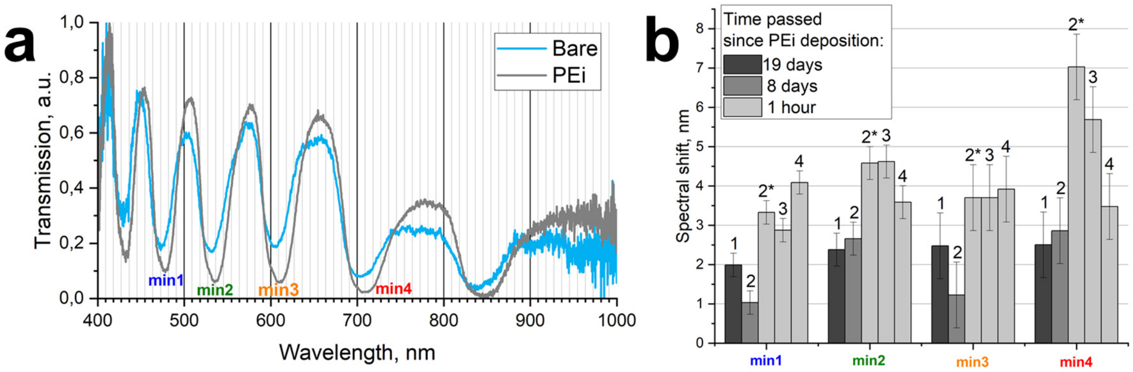

Figure S3. Shifts in transmission spectra induced by PEi: (a) transmission spectra of the same HC-MOF before and after PEi deposition, with positions of minima selected for analysis labeled as min1...min4 ; (b) shifts in transmission spectra induced by PEi to a set of HC-MOFs.
